# Postabortion contraceptive use in Bahir Dar, Ethiopia: a cross sectional study

**DOI:** 10.1186/s40834-019-0099-8

**Published:** 2019-11-04

**Authors:** Anteneh Mekuria, Hordofa Gutema, Habtamu Wondiye, Million Abera

**Affiliations:** 1 Marie Stopes International Ethiopia, Bahir Dar Maternal and Child Health center, Bahir Dar, Ethiopia; 2 Department of Health Promotion and Behavioral Sciences, School of Public Health, College of Medicine and Health Sciences, Bahir Dar University, Bahir Dar, Ethiopia; 3 School of Nursing and Midwifery, Institute of Health, Jimma University, Jimma, Ethiopia

**Keywords:** Postabortion contraceptive use, Associated factor, Cross sectional study, Bahir Dar

## Abstract

**Background:**

Although promoting postabortion family planning is very important and effective strategy to avert unwanted pregnancy, less attention was given to it in Ethiopia. Thus, this study aimed to assess contraceptive use and factors which are affecting it among women after abortion in Bahir Dar town.

**Methods:**

Facility based cross-sectional study was conducted in Bahir Dar town. The data was collected using structured interviewer administered questionnaire from women who obtain the abortion services. Bivariable and multivariable logistic regression was used to evaluate the association that demographic factor and reproductive characteristics have with postabortion contracetive use. Findings with *p*-value of < 0.05 at 95% CI were considered as statistically significant.

**Results:**

A total of 400 women who received abortion service were participated in this study. The proportion of postabortion contraceptive use is 78.5%. Single women are 7.2 times more likely use contraceptive after abortion as compared to their counterpart. Contraceptive use is 2 times higher among women who have previous history of abortion as compared to their counterpart. Women who used contraceptive previously and who used contraception for index pregnancy are 4.73 and 2.64 times more likely to use contraceptive after abortion as compared to their counterpart respectively.

**Conclusion:**

Postabortion contraceptive use is associated with age, marital status, having previous history of abortion, previous contraceptive use and using contraception for index pregnancy. Greater emphasis should be given on providing postabortion contraceptive counselling to increase utilization of postabortion contraceptive use.

## Background

Postabortion family planning (PAFP) is a key component of Postabortion care (PAC); and it includes voluntary contraceptive counseling and service provision [1]. World Health Organization (WHO) recommends that Women can to start hormonal contraception at the time of surgical abortion and an IUD can be inserted when it is reasonably certain that the woman is no longer pregnant following medical abortion [2]. Voluntary family planning counseling and services should be offered immediately after abortion at the site of care to reduce unintended pregnancies and repeat abortions and to reduce the risks of adverse maternal and perinatal outcomes for pregnancies following induced abortion [3].

Globally, it is estimated that there are 80 million of women experience unintended pregnancy. A Of these about 44 million of them have induced abortion [4] and of these women 22 million of them are undergoing unsafe abortion; which is the reason for death of 47, 000 women [5]. Women who have an induced abortion have had a previous abortion most of the time [6], yet many of these women do not have access to effective contraceptives and are not offered immediate post abortion family planning services, even though women who receive abortion care are at risk of pregnancy immediately after the procedure [3].

In developing countries, about 885 million of reproductive age (15–49) want to prevent becoming pregnant; of these women, about 671 million of them are using contraceptive while the remaining 214 million of them are considered to have unmet need of contraceptives and the highest proportion (21%) of unmet need for contraception is observed in Sub-Saharan Africa [7]. Such low contraceptive rate and high unmet need for family planning give rise to high rate of unintended pregnancies and unsafe abortion.

Although contraceptive acceptance rate has improved in the last decade from 8.2 to 28.6% in Ethiopia, it remains as one of the countries with low contraceptive use and unmet need for family planning is 25.3% [8]. In Ethiopia, it is legal for a women to terminate a pregnancy if her life is in danger, if she has physical or mental disabilities, or if she is a minor who is physically or mentally unprepared for childbirth. Additionally, abortion is legal in cases of incest, fetal impairment or rape [9].

Factor like lack of access to effective contraceptives, not offering immediate PAFP service, inconsistent use of short acting contraceptive and method failure are reported as cause of high burden of unintended pregnancies and recurrent abortion [7]. Even though PAFP has important impact to reduce recurrence of unintended pregnancies and abortion, it is not given due attention in Ethiopia. Failing to give attention to PAFP will result with repeated abortion and unintended pregnancies as women who received the abortion care leave health facility without family planning service [6].

Indeed, plenty of studies were conducted on PAFP in Ethiopia, but not only the finding in proportion of PAFP utilization, their study design, objectives of the study, targeted population and the selected facility were different among them, and variables like previous history of abortion were not assessed in some of the studies [8, 10–13] and we urgue that these findings are not representing the target group in Bahir Dar city. Hence, the aim of this study to assess postabortion contraceptive use and associated factors among women of reproductive age group in Bahir Dar town.

## Methods

### Study design and setting

We performed a facility-based cross-sectional study was conducted in health facilities providing Postabortion service in Bahir Dar Town. Bahir Dar town is located in Northwest part of the Ethiopia and it is 570 km from Addis Ababa. A total of 288,200 people live in the town. Women in reproductive age group account about 23.6% of the total. The town has one referral hospital, four health centers and two non-governmental reproductive health clinics and contraception is provided for no cost in all health facilities.

### Study participants

Women in reproductive age group (15–49 years) and all women who were attending the selected health facilities for safe abortion service during the period October 1 – November 1, 2015 were included. Participant who were unable to provide information due to severe illness and those with abortion complication like excessive bleeding were excluded.

### Sampling

Sample size was calculated using single population proportion formula by assuming proportion of postabortion modern contraceptive use to be 57% [10], confidence level of 95% (z α/2 = 1.96) and 5% margin of error. The final sample size was determined to 415 after adding 10% non-response rate. All the seven health facilities (two non-governmental clinics- which were ran by Marie Stopes International and Ethiopian Family Guidance Association, one public hospital and four public health centers) which were giving family planning services in Bahir Dar city were included in the study. Sample size for each health facility was determined after preliminary assessment of past two months’ comprehensive abortion care and using proportional allocation technique to size. The number of participant assigned for the public hospital was 94 individual and 53 participants for four of the health center, while 243 participant where assigned for the clinic which is ran by Marie Stopes and 25 participant assigned for the clinic which ran by Family Guidance Ethiopia. All of the women who seek for the abortion service in health facilities were included consecutively until the required sample was fulfilled.

### Data collection procedure

Data was collected by using interviewer administered structured questionnaire adopted from handbook for measuring and assessing the Integration of Family Planning and Other Reproductive Health Services after modifying it to the study context [14]. The questionnaire includes sociodemographic factors such as age, marital status, educational status, religion, occupation and residence; and reproductive factors like contraceptive history, history of birth, future fertility preference, past abortion history, contraceptive use with index pregnancy. The questionnaire was initially developed in English and translated to local language Amharic and back translated to English by language expert to check for consistency of meaning. We trained six female nurse to perform data collection and one health officer was recruited to supervise the data collection process. Pre-testing was also conducted on 5% of the sample size outside of the study area prior to actual data collection to check for unclear information and to make modification based on its finding. All the participants were reached through exit interview after the women get abortion service at selected health facilities and the interview was conducted in private place in the facility.

### Data analysis

Data was edited and entered into Epi Info version 3.5.3 and then exported to SPSS version 20 for analysis. Descriptive statistics and summary measures of the variables were done. Chi square was used as to check the association between postabortion contraceptive use and independent variables. Multiple logistic regression analysis was used for evaluation of postabortion contraceptive use. All variables with *p*-value < 0.2 in the bivariate analysis were entered in to the multivariable logistic regression analysis. Adjusted odds ratio (AOR) with 95% confidence interval (CI) was used to identify the independent predictors of PAFP. To claim statistically significant effect, crude and adjusted odds ratio with 95% (CI) was employed at *p* value < 0.05.

## Result

### Socio demographic characteristics

We approached 415 women for interview but 400 of them were participated in the study and it make the response rate of 96.4% while those remaining 15 women were refused to participate. One hundred sixty three (40.8%) of the respondents were in the age group 20–24 with mean age of 24.2 (SD ±4.9) years. Three hundred twenty-six (81.7%) of respondents were married and two hundred thirty-seven (59.2%) of them completed secondary school and above. Above two third (73.5%) of study subjects were urban dwellers (Table 1).

**Table 1:** Socio demographic characteristics of women of reproductive age group (15–49) who received abortion care in Bahir Dar Town, October 1 – November 12,015, *n* = 400

Variables	Postabortion contraceptive		*P*-value
	Yes n (%)	No n (%)	
Age			
15–19	47 (15.3%)	13 (14.1%)	0.17
20–24	126 (40.9%)	37 (40.2%)	
25–29	97 (31.5%)	18 (19.5%)	
30–34	23 (7.5%)	10 (10.9%)	
≥ 35	15 (4.8%)	14 (15.3%)	
Marital status			
Married	275 (89.3%)	51 (55.4%)	< 0.000
Single	33 (10.7%)	41 (44.6%)	
Educational status			
No formal education	61 (19.4%)	12 (13.9%)	0.198
Primary school	68 (21.7%)	22 (25.6%)	
Secondary school and above	185 (58.9%)	52 (60.5%)	
Occupational status			
Employed	137 (43.6%)	32 (37.2%)	0.35
Homemaker	85 (27.1%)	22 (25.6%)	
Student	92 (29.3%)	32 (37.2%)	
Religion			
Orthodox	289 (91.8%)	75 (88.2%)	0.64
Muslim	23 (7.3%)	8 (9.4%)	
Others *	3 (0.9%)	2 (2.4%)	
Residence			
Urban	233 (74.2%)	61 (71%)	0.37
Rural	81 (25.8%)	25 (29%)	
Have children			
Yes	140 (44.6%)	29 (33.7%)	0.27
No	174 (55.4%)	57 (66.3%)	

*= Protestant and Catholic

### Reproductive characteristics

One hundred six (26.5%) of women had previous history of abortion. Large number (92.5%) of respondent had no desire for current pregnancy. Two hundred ninety- one (72.8%) of them had history of taking contraceptive. One hundred thirty-seven (34.2%) of the study subjects used contraceptive during the occurrence of the index pregnancy. Three hundred sixty-six (91.5%) of the women have a desire to be pregnant again.

About three fourth (78.5%) of them used family planning method after current abortion. Of those who use family planning method, around two third (64%) of them used long term contraceptive mothed. Only around one third (31.8%) of the study participant were informed about the time period that they become pregnant again following the abortion. Above four from five (81.75%) of the participants had gestational age of less than 12 weeks by the time they receive abortion service (Table 2).

**Table 2:** Reproductive condition distribution of women of reproductive age group (15–49) who received abortion care in Bahir Dar Town, October 1 – November 1, 2015, *n* = 400

Variables	Postabortion contraceptive		*p* value
	Yes n (%)	No n (%)	
Previous abortion history			
Yes	83 (26.4%)	23 (26.7%)	0.15
No	231 (73.6%)	63 (73.3%)	
Have the desire for current pregnancy			
Yes	25 (8%)	5 (5.8%)	0.50
No	289 (92%)	81 (94.2%)	
Ever used contraceptive before			
Yes	250 (79.6%)	41 (47.7%)	< 0.000
No	64 (20.4%)	45 (52.3%)	
Method ever used			
Short acting	226 (90.4%)	35 (85.4%)	0.32
Long acting	24 (9.6%)	6 (14.6%)	
Desire to have a child in the future			
Yes	285 (90.8%)	81 (94.2%)	0.31
No	29 (9.2%)	5 (5.8%)	
Used contraceptive during the index pregnancy			
Yes	126 (40.1%)	11 (12.8%)	< 0.000
No	188 (59.9%)	75 (87.2%)	
Method used during the current pregnancy			
Pills	105 (83.3%)	9 (81.8%)	0.24
Injectable	19 (15.1%)	1 (9.1%)	
Condom	2 (1.6%)	1 (9.1%)	
Current mode of termination			
Medical	223 (71%)	69 (80.2%)	0.28
Surgical	91 (29%)	17 (19.8%)	
Informed how soon become pregnant			
Yes	101 (32.2%)	26 (30.2%)	0.73
No	213 (67.8%)	60 (69.8%)	
Gestational Age			
≤ 12	248 (79%)	79 (91.9%)	0.35
> 12	66 (21%)	7 (8.1%)	

### Independent predictors of postabortion contraceptive use

Variables which were significantly association with postabortion contraceptive use in the bivariate analysis at *p* value less than 0.2 were entered into the final model. As shown in Table 3, postabortion contraceptive use was 8.52, 5.08, 5.76 and 4.2 times higher among women in age group 20–24, 25–29, 30–34; and 35 and above years as compared with those who are aged between 15 and 19 years [AOR = 8.52, 95% CI: 2.6–17.92, AOR = 5.08, 95% CI: 1.91–13.5, AOR = 5.76, 95% CI:2.11–15.7 and AOR = 4.2, 95% CI:1.29–13.63] respectively. Single women were 7.2 times more likely to use contraceptive after abortion as compared with married women [AOR = 7.2, 95% CI: 3.89–13.33]. Postabortion contraceptive use was 2 times higher among those who have previous history of abortion as compared with those who do not have experienced it [AOR = 2.0, 95% CI: 1.23–3.84].

**Table 3:** Predictors of postabortion contraceptive use among women of reproductive age group (15–49) who received abortion care in Bahir Dar Town, October 1–November 1, 2015, *n* = 400

Variables	Postabortion contraceptive		Odd ratio (95% CI)	
	Yes n (%)	No n (%)	Crude	Adjusted
Age				
15–19	47 (15.3%)	13 (14.1%)	1	1
20–24	126 (40.9%)	37 (40.2%)	3.37 (1.30–8.74)	8.52 (2.6–17.92)*
25–29	97 (31.5%)	18 (19.5%)	3.17 (1.40–7.18)	5.08 (1.91–13.5) *
30–34	23 (7.5%)	10 (10.9%)	5.03 (2.24–1.66)	5.76 (2.11–15.7)*
≥ 35	15 (4.8%)	14 (15.3%)	2.14 (0.76–6.07)	4.20 (1.29–13.63)*
Marital status				
Married	275 (89.3%)	51 (55.4%)	1	1
Single	33 (10.7%)	41 (44.6%)	6.7 (3.87–11.57)*	7.2 (3.89–13.33) *
Previous abortion history				
No	231 (73.6%)	63 (73.3%)	1	1
Yes	83 (26.4%)	23 (26.7%)	1.29 (0.46–1.30)	2.00 (1.23–3.84)*
Ever used contraceptive				
No	64 (20.4%)	45 (52.3%)	1	**1**
Yes	250 (79.6%)	41 (47.7. 2%)	3.65 (2.23–5.98)*	4.73 (2.40–9.30)*
Used contraceptive during the index pregnancy				
No	188 (59.9%)	75 (87.2%)	1	1
Yes	126 (40.1%)	11 (12.8%)	3.07 (1.71–5.52) *	2.64 (1.82–3.31) *

*significant at *p* < 0.05

Postabortion contraceptive use was 4.73 times higher among women who ever used contraceptive as compared with who never used [AOR = 4.73, 95% CI: 2.4–93]. Those who used any contraceptive method during the index pregnancy were 2.64 times more likely use contraceptive after abortion as compared with women who do not used [AOR = 2.64, 95% CI: 1.82–3.31].

## Discussion

In this study, 78.5% of women had used contraceptive after receiving abortion care. This finding is high when compared to studies conducted in two different parts of the country. In study conducted in health facilities in Amhara and Oromia regions, the contraceptive uptake was 44.7% [12]. The finding of the study performed among mother who receive abortion service in Dessie town has shown 47.5% of contraceptive [8]. Considering only women who received abortion care from public facility where there is shortage contraception supplies in study conducted in two of the region and failing to integrate post abortion family planning service within post abortion care in dessie might be the reason for this discripancy However, the finding of the study performed in turkey is also comparable indicating 78% of women use at least one contraceptive method after abortion [15].

Of those who used contraceptive, 64% of them used long term contraceptive. This is comparable with finding of study conducted in Turkey has shown that 52.3% of women who never used Intra Uterine Device (IUD) has started using it during postabortion care [15]. Becoming pregnant while using short term contraceptive (34.2%) of the participants might be reason for prefering long acting contraceptive method. However, this finding is very different from those of studies conducted in Dar es salaam, Tanzania (68%) and Brazil (84.3%) women preferred using short acting contraceptive such as pills and injectable [16, 17].

Majority (92.5%) of participants, the current pregnancy which end up with abortion was not wanted. This is comparable with the finding of the study in Dessie, the index pregnancy was unwanted for 92.2% of participants [8]. The reasons for ending up the pregnancy for women who wanted it inititally were spontaneous occurance of abortion, partner pressure and other health problem.

Women in age group 20–24, 25–29, 30–34; and 35 and above years were 8.52, 5.08, 5.76 and 4.2 times more likely use postabortion contraceptive as compared with those who aged between 15 and 19 years. The finding of previous studies also showed that the increase in likelihood of contraception use as age of the women who receive abortion care increased [11, 18, 19]. Single women were 7.2 times more likely to use contraceptive after abortion as compared with married women. Married women might be under influence of their partner and it implies the importance engagement of male partner in postabortion contraceptive use counseling to increase its uptake. Postabortion contraceptive use was 2 times higher among women who have previous history of abortion as compared with those who do not have previous abortion history. This finding is similar with study performed in Addis Ababa in which previous history of abortion was significantly associated with postabortion adoption of modern contraception [20].

Postabortion contraceptive use was 4.73 times higher among women who ever used contraceptive compared to who never used any contraception. This is in line with study done in Addis Ababa and Pakistan in which women who ever used contraception were 2 and 1.22 times more likely used contraception after abortion compared to those who never used it respectively [20, 21]. Women who never used contraceptive should be given detail information and councelling on the contraception when they visit health facility for abortion services. Those women who have used contraceptive during the index pregnancy were 2.64 times more likely to use contraceptive after abortion as compared with their counterpart. Study done in Dessie is comparable with the current finding, in which women who used contraceptive method during the index pregnancy were 2.3 times more likely use contraception postabortion compared to who didn’t use it for index pregnancy [8]. A major limitation of this study was that cross-sectional design was used and therefore we cannot report cause and effect. Future study should examine the causal relationship of the variables using analytical study design.

## Conclusion

The finding of this study indicated relatively high utilization of contraceptive method after abortion. Age, marital status, previous history of abortion, ever use of contraceptive and using contraceptive for index pregnancy were significantly associated with postabortion contraceptive use. Greater emphasis should be given on providing postabortion contraceptive counselling, particularly for younger, married, and those who do not have previous abortion history, never used contraception and used it for current pregnancy, to increase utilization of postabortion contraceptive use.

## Data Availability

The datasets used and analysed during the current study are available from the corresponding author on reasonable request.

## Abbreviations

CI: Confidence Interval
FP: Family planning
IUD: Intrauterine device
NGOs: Nongovernmental organizations
OR: Odd Ratio
PAC: Postabortion care
PAFP: Postabortion family planning
SD: Standard deviation
WHO: World Health Organization
